# K^+^-Dependent Na^+^/Ca^2+^ Exchanger Isoform 2, Nckx2, Takes Part in the Neuroprotection Elicited by Ischemic Preconditioning in Brain Ischemia

**DOI:** 10.3390/ijms23137128

**Published:** 2022-06-27

**Authors:** Ornella Cuomo, Rossana Sirabella, Francesca Boscia, Antonella Casamassa, Jonathan Lytton, Lucio Annunziato, Giuseppe Pignataro

**Affiliations:** 1 Division of Pharmacology, Department of Neuroscience, School of Medicine, University of Naples Federico II, 80131 Naples, Italy; ornella.cuomo@unina.it (O.C.); rossana.sirabella@unina.it (R.S.); boscia@unina.it (F.B.); 2 IRCCS SYNLAB SDN S.p.A., 80143 Naples, Italy; antonella.casamassa@unina.it; 3 Cardiovascular Research Group, Department of Biochemistry and Molecular Biology, University of Calgary, Calgary, AB T2N 4N1, Canada; jlytton@ucalgary.ca

**Keywords:** ischemic preconditioning, neuroprotection, sodium/calcium exchanger, ionic homeostasis

## Abstract

Sodium/Calcium exchangers are neuronal plasma membrane antiporters which, by coupling Ca^2+^ and Na^+^ fluxes across neuronal membranes, play a relevant role in brain ischemia. The most brain-expressed isoform among the members of the K^+^-dependent Na^+^/Ca^2+^ exchanger family, NCKX2, is involved in the progression of the ischemic lesion, since both its knocking-down and its knocking-out worsens ischemic damage. The aim of this study was to elucidate whether NCKX2 functions as an effector in the neuroprotection evoked by ischemic preconditioning. For this purpose, we investigated: (1) brain NCKX2 expression after preconditioning and preconditioning + ischemia; (2) the contribution of AKT and calpain to modulating NCKX2 expression during preconditioning; and (3) the effect of NCKX2 knocking-out on the neuroprotection mediated by ischemic preconditioning. Our results showed that NCKX2 expression increased in those brain regions protected by ischemic preconditioning. These changes were p-AKT-mediated since its inhibition prevented NCKX2 up-regulation. More interestingly, NCKX2 knocking-out significantly prevented the protection exerted by ischemic preconditioning. Overall, our results suggest that NCKX2 plays a fundamental role in the neuroprotective effect mediated by ischemic preconditioning and support the idea that the enhancement of its expression and activity might represent a reasonable strategy to reduce infarct extension after stroke.

## 1. Introduction

Since the failure of multiple clinical trials of potential stroke neuroprotectants [1], attention has turned to the identification of new strategies of intervention. In this attempt, ischemic conditioning represents a promising strategy for the identification of protective biochemical pathways and for the validation of druggable targets. In fact, the neuroprotective concept of preconditioning is based on the observation that a brief, non-injurious episode of ischemia is enough to trigger a genomic response able to protect the brain from a subsequent longer ischemic insult [2,3,4]. Although several pathways have been proposed as plausible mechanisms involved in the neuroprotection offered by ischemic preconditioning [5,6], molecular mechanisms of neuroprotection that lead to ischaemic tolerance are incompletely understood. Identification of genes involved in this process would provide insight into cell survival and innovative therapeutic approaches for stroke.

In the last decade, growing evidence has shown that proteins involved in the maintenance of ionic homeostasis play a fundamental role in stroke pathophysiology and in brain conditioning neuroprotection [7,8]. In particular, among the members of the superfamily of cation/Ca^2+^ plasma membrane exchangers, the isoform 3 of the K^+^-independent Na^+^-Ca^2+^ exchangers contributes to the neuroprotection induced by pre- and postconditioning since its knocking-down, induced by intracerebroventricular infusion of a siRNA against NCX3, or its genetic disruption (ncx3−/− mice) partially prevented the preconditioning and postconditioning-induced neuroprotection [9,10]. Moreover, NCX3 upregulation was p-AKT-mediated, since it was prevented by its inhibition, as it has been demonstrated that NCX3 is an additional target for the prosurvival action of Akt [11]. Interestingly, NCKX2, the isoform most expressed in the brain among the members of the family of the K^+^-dependent Na^+^/Ca^2+^ exchangers (NCKX), exerts a relevant role during cerebral ischemia since both its knocking-down and its knocking-out dramatically increase the extent of the ischemic lesion in rats and mice subjected to brain ischemia. Moreover, focal cerebral ischemia caused relevant changes in the pattern of both NCKX2 mRNA and protein expression in the ischemic core and in the remaining ipsilateral non-ischemic area [12]. These findings indicate that the disruption of the nckx2 gene by genetic manipulation renders neurons more susceptible to the ischemic insult and that NCKX2 activity attenuates the development of brain injury elicited by ischemia. In the light of the above-described results, highlighting a key role of NCKX2 during cerebral ischemia, the aims of this study were: (a) to evaluate whether NCKX2 intervenes in preconditioning-elicited neuroprotection and (b) to clarify whether modifications in NCKX2 expression occurring during this neuroprotective strategy depend on AKT or on calpain-dependent post-translational pathways.

To this aim, we evaluated: (1) brain NCKX2 expression after preconditioning and preconditioning + ischemia; (2) the effect of AKT inhibition on NCKX2 expression during preconditioning; (3) the activation of calpain and of calpain inhibition on NCKX2 expression after ischemia and preconditioning; (4) the effect of NCKX2 knocking-out on the neuroprotection mediated by ischemic preconditioning.

## 2. Results

### 2.1. Ischemic Preconditioning Induces NCKX2 Overexpression in the Peri-Ischemic Temporoparietal Cortex and Prevents NCKX2 Downregulation in the Striatum of Ischemic Rats

**Figure 1:**
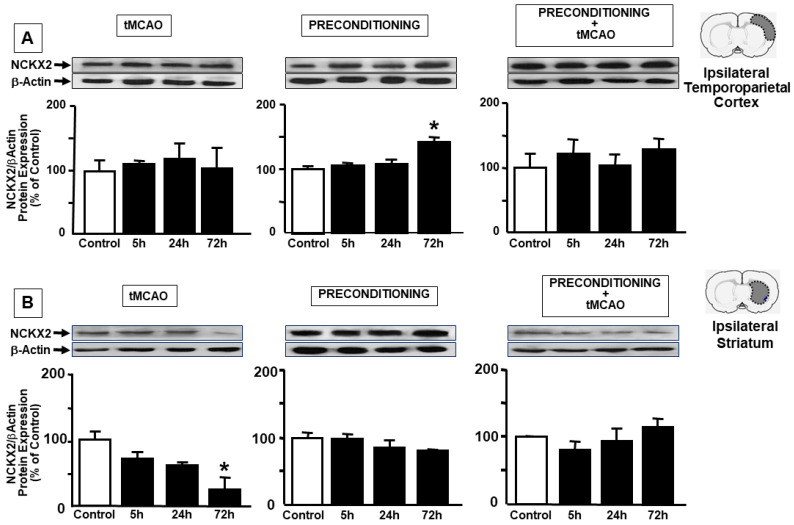
**NCKX2 PROTEIN EXPRESSION IN RAT IPSILATERAL CORTEX AND STRIATUM AFTER tMCAO, PRECONDITIONING, AND PRECONDITIONING + tMCAO** Time course of NCKX2 protein expression after tMCAO, preconditioning and preconditioning + tMCAO in the ipsilateral temporoparietal cortex (panel **A**) and in the striatum (panel **B**). A representative brain slice cartoon indicating the area of interest is on the top of the panels of the figure. Data were normalized on the basis of β-actin levels and expressed as a percentage of control animals. On the x-axis is the reperfusion time interval. Values are mean± SEM. * *p* < 0.05, compared with control. n = 5 animals for each column.

In order to assess the role played by NCKX2 during preconditioning, its expression was evaluated in the ipsilesional temporoparietal cortex and in the striatum of rats exposed to ischemia, preconditioning stimulus and preconditioning plus ischemia. Rats were sacrificed at the following reperfusion time intervals: 5 h, 24 h and 72 h, in order to include the entire temporal window of the development of brain ischemic damage. Immunoblot analysis from peri-ischemic temporoparietal cortex lysates showed a significant increase in NCKX2 protein levels 72 h after preconditioning, while no significant changes were observed in ischemia and preconditioning plus ischemia experimental groups at any time point analyzed (Figure 1A). Interestingly, in the striatum, the dramatic decrease in NCKX2 expression occurring after 100′ of transient middle cerebral artery occlusion (tMCAO), was not observed both in the preconditioning and preconditioning plus ischemia experimental groups at any time point analyzed (Figure 1B).

**Figure 2:**
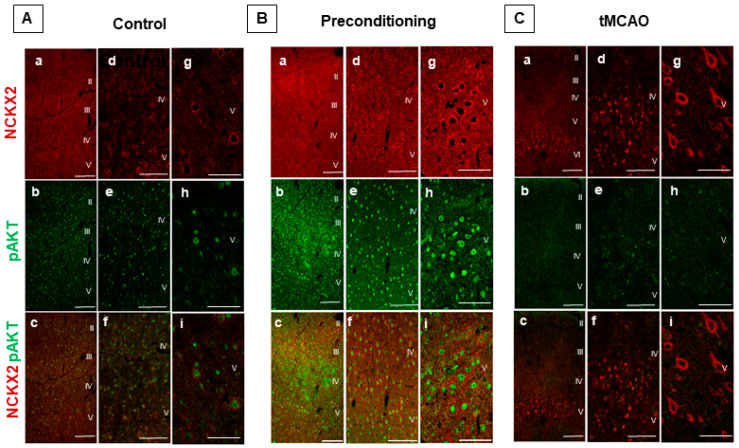
**COEXPRESSION OF NCKX2 AND pAKT IMMUNOREACTIVITIES IN THE PERIISCHEMIC CORTICAL REGION OF PRECONDITIONED AND ISCHEMIC RATS.** (**A**–**C**), Representative images displaying the co-expression of NCKX2 with pAKT immunoreactivities in the temporoparietal cortex of control (**A**), preconditioned (**B**) and ischemic animals (**C**). Reperfusion time was 72 h both in preconditioned and ischemic animals. Panels a–c, d–f and g–i were acquired at increasing magnification of 10×, 20× and 50×, respectively. Higher magnification images were taken in the deep cortical layers. Scale bars in a–c: 200 μm, in d–f: 100 μm, in g–i: 50 μm.

In particular, 72 h after tMCAO, we performed confocal colocalization analyses to investigate the co-expression of NCKX2 with p-AKT in our experimental conditions, as p-AKT is a well-known transducer of preconditioning induced neuroprotection. As expected, in control conditions, the anti-NCKX2 antibody revealed a more intense distribution of the exchanger in neurons of the deep cortical layers (IV-V), and a moderate nuclear pAKT staining throughout all cortical layers (Figure 2A). Co-expression analysis revealed that NCKX2 and the nuclear pAKT immunoreactivities concomitantly increased 72 h after the preconditioning stimulus, particularly in neurons of the deep IV-V cortical layers (Figure 2B). Conversely, NCKX2 and pAKT co-expression intensely decreased in the ipsilateral temporoparietal cortex of ischemic rats 72 h after tMCAO. Nevertheless, in these experimental conditions, several intensely stained NCKX2-positive cells were still observed at the perilesional border of the ischemic region (Figure 2C).

**Figure 3:**
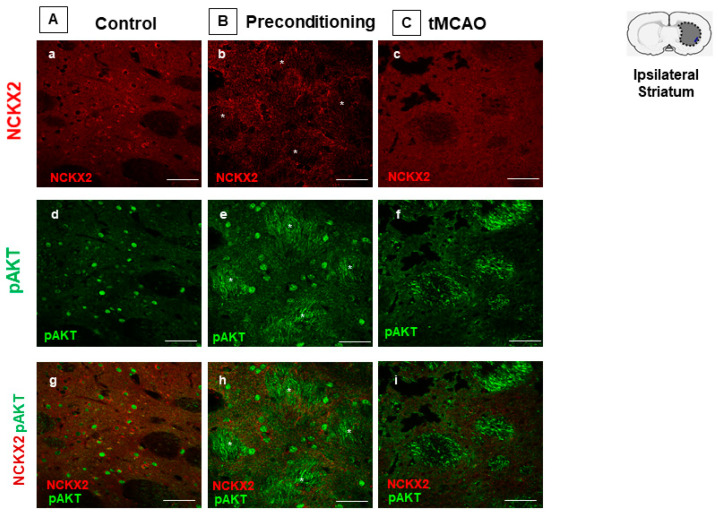
**CO-EXPRESSION OF NCKX2 AND pAKT IMMUNOREACTIVITIES IN THE STRIATUM OF PRECONDITIONED AND ISCHEMIC RATS.** Representative images (g–i) displaying the co-expression of NCKX2 (a–c) with pAKT (d–f) immunoreactivities in the striatum of control (**A**), preconditioned (**B**) and ischemic animals (**C**). Scale bars 50 μm.

As observed in the temporoparietal cortex, the preconditioning stimulus intensely increased pAKT, but not NCKX2 signal in the striatum. In this region, pAKT was clearly detected in fiber bundles, in which NCKX2 was not detected (Figure 3).

### 2.2. p-AKT Inhibitor LY294002 Prevents NCKX2 Overexpression Induced by Preconditioning in the Peri-Ischemic Temporoparietal Cortex

**Figure 4:**
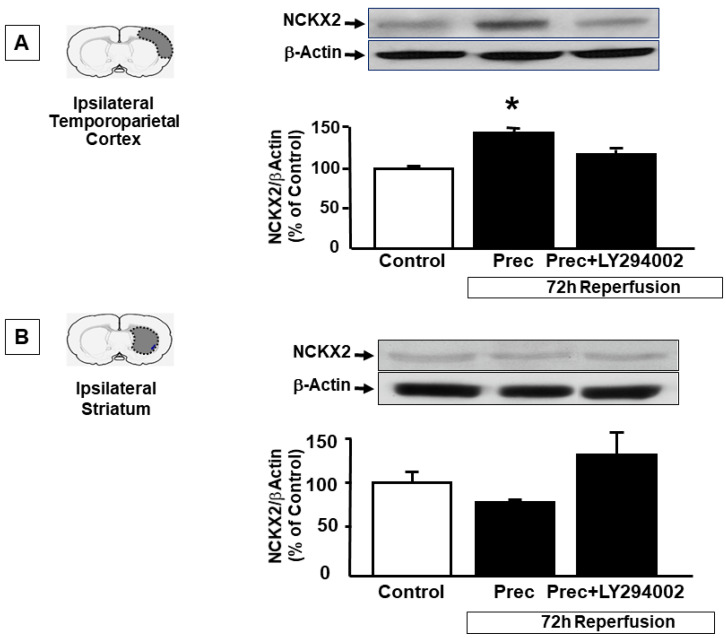
**EFFECT OF THE pAKT INHIBITOR LY2940002 ON NCKX2 EXPRESSION DURING PRECONDITIONING IN CORTEX AND STRIATUM.** (**A**) Western blot analysis of NCKX2 protein levels in the temporoparietal cortex of sham-operated animals, rats subjected to preconditioning and treated with vehicle or rats treated with the pAKT inhibitor LY294002 and then subjected to preconditioning stimulus. All the animals were sacrificed 72 h after the surgery. (**B**) Western blot analysis of NCKX2 protein levels in the ipsilateral striatum of sham-operated animals, rats subjected to tMCAO and treated with vehicle or rats treated with the pAKT inhibitor LY294002 and then subjected to preconditioning stimulus. All the animals were sacrificed 72 h after the surgery. Values are mean ± SEM. * *p* < 0.05, compared with control group. n = 3–5 animals for each column.

To further support the tight interaction between NCKX2 and p-AKT, the p-AKT inhibitor LY294002 was icv injected before preconditioning induction and NCKX2 expression was evaluated in the ipsilesional temporoparietal cortex and in the striatum of rats exposed to ischemic preconditioning at 72 h of reperfusion and compared to the expression of the protein in the same brain regions of vehicle-treated rats. In accordance with our results from double immunofluorescence experiments, showing a co-expression of NCX2 and pAKT in this brain region, the increase in NCKX2 protein expression induced by preconditioning in the temporoparietal cortex was prevented by the p-AKT inhibitor LY294002 (143 ± 8 vs. 117 ± 5.5, respectively) (Figure 4A), thus suggesting that NCKX2 overexpression induced by preconditioning in the cortex may occur through a p-AKT-mediated translational activation. By contrast, as expected, in the striatum, the use of LY294002 did not modify NCKX2 expression (Figure 4B), thus supporting the hypothesis that in this area and in these experimental conditions, NCKX2 synthesis did not increase, but rather it prevented its degradation promoted by the lethal ischemic insult. This result was in line with immunofluorescence experiments showing that after preconditioning, pAKT did not colocalize with NCKX2 in the striatum (Figure 3).

### 2.3. Preconditioning Prevents Calpain Activation Responsible for NCKX2 Downregulation in the Striatum of Ischemic Rats

**Figure 5:**
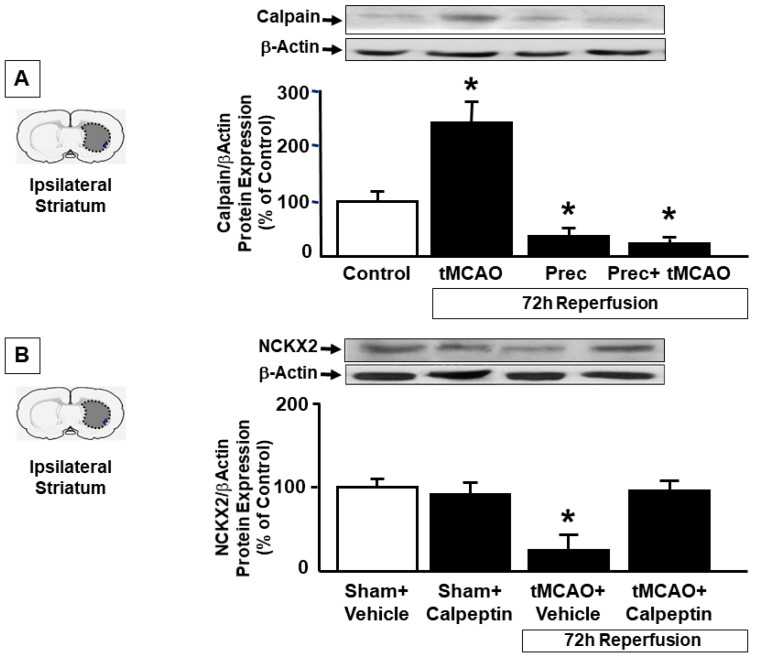
**CALPAIN ACTIVATION DURING ISCHEMIA AND PRECONDITIONING AND EFFECT OF CALPEPTIN ON NCKX2 EXPRESSION AFTER ISCHEMIA.** (**A**) Western blot analysis of calpain protein levels in the ipsilateral striatum of sham-operated animals, rats subjected to tMCAO, preconditioning or preconditioning + tMCAO. All the animals were sacrificed 72 h after the surgery. (**B**) Western blot analysis of NCKX2 protein levels in the ipsilateral striatum of sham-operated rats receiving vehicle, sham-operated rats receiving calpeptin, ischemic rats treated with vehicle or with calpeptin. All the animals were sacrificed 72 h after the surgery. Values are mean ± SEM. * *p* < 0.05, compared with control group. n = 3–5 animals for each column.

As AKT inhibition did not modify striatal NCKX2 expression after preconditioning, we hypothesized that preconditioning was able to prevent the downregulation of NCKX2 occurring during ischemia by inhibiting its degradation. Since we have previously demonstrated that, in a model of permanent focal ischemia, NCKX2 downregulation after brain ischemia was mediated by calpain, we measured calpain activation in the striatum of rats exposed to tMCAO, preconditioning and preconditioning plus tMCAO. We found that 72 h after tMCAO, calpain was dramatically activated, and this activation was completely prevented by preconditioning stimulus (Figure 5A), thus supporting the idea that during preconditioning, NCKX2 is no more degraded.

To confirm that NCKX2 downregulation observed 72 h after tMCAO in the striatum was due to protein degradation, calpeptin was icv administered as previously described and NCKX2 expression was evaluated 72 h after tMCAO induction. Western Blot experiments showed that NCKX2 downregulation occurring after tMCAO was completely prevented in rats pre-treated with calpeptin (Figure 5B), thus suggesting that after ischemia the decrease in NCKX2 expression was due to a calpain-mediated degradation, and that preconditioning prevented calpain-induced NCKX2 degradation. No changes in NCKX2 expression after calpeptin treatment was observed in sham-operated animals (data not shown).

### 2.4. NCKX2 Knocking-out Prevents the Neuroprotective Effect on Brain Ischemia Induced by Ischemic Preconditioning

**Figure 6:**
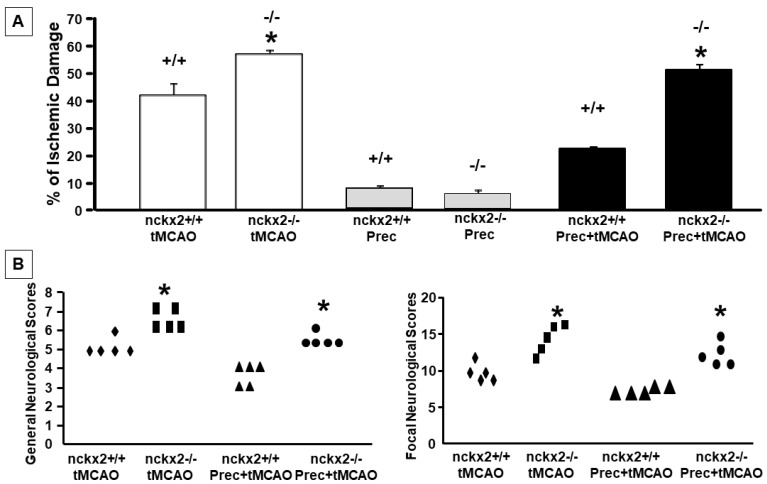
**EFFECT OF NCKX2 KNOCKING-OUT ON THE NEUROPROTECTION MEDIATED BY PRECONDITIONING.** (**A**) Percentage of the Ischemic Damage in nckx2+/+ and nckx2−/− mice subjected to tMCAO, preconditioning or preconditioning + tMCAO. Mice were euthanized 24 h after surgery. * *p* < 0.05 versus tMCAO group. (**B**) General and Focal Neurological scores in nckx2+/+ and nckx2−/− mice subjected to tMCAO or tMCAO + preconditioning. Neurological function was assessed 24 h after surgery. * *p* < 0.05 versus tMCAO group.

In order to assess whether the changes observed in NCKX2 expression had a pathophysiological meaning, nckx2+/+ and nckx2−/− mice were subjected to tMCAO, or to preconditioning plus tMCAO and the infarct volume was evaluated 24 h after surgery. Our results showed that NCKX2 knocking-out was able to induce a loss of the neuroprotective effect induced by preconditioning (% infarct volume: 42.3 ± 6.6 in nckx2+/+ mice subjected to tMCAO, 57.3 ± 1.2 in nckx2−/− subjected to tMCAO, 22.4 ± 0.5 in nckx2+/+ subjected to preconditioning + tMCAO, 51.6 ± 2.1 in nckx2−/− subjected to preconditioning + tMCAO) (Figure 6A). In parallel with the loss of preconditioning-induced brain protection, no improvement in neurological functions was assessed after preconditioning plus tMCAO in nckx2−/− mice (Figure 6B). No differences were found between nckx2−/− and ncxkx2+/+ animals exposed to preconditioning alone (Figure 6A).

## 3. Discussion

The results shown in the present paper demonstrate for the first time that NCKX2, the isoform most expressed in the brain among the members of the family of the K^+^-dependent Na^+^/Ca^2+^ exchangers, represents an additional new molecular effector involved in the neuroprotection exerted by ischemic preconditioning.

In particular, we showed that preconditioning alone induced an upregulation of NCKX2 expression in the brain cortex but not in the striatum and prevented tMCAO-induced NCKX2 reduction in the striatum.

A possible explanation for NCKX2 upregulation after preconditioning in the cortex is provided thanks to our results showing that during preconditioning the increase of NCKX2 levels in the temporoparietal cortex is p-AKT-mediated. In fact, p-AKT inhibition induced by LY294002 prevented NCKX2 upregulation. In further support of this hypothesis is the observation that, after preconditioning, NCKX2 and pAKT immunosignals increase and strongly colocalize in the brain cortex, particularly in neurons of the deep IV-V cortical layers. By contrast, the observation that preconditioning in the striatum does not induce an increased expression but rather prevents the dramatic NCKX2 downregulation occurring after cerebral ischemia might be explained by the fact that the striatum represents a brain region strongly affected by the ischemic damage after tMCAO and the only brain region damaged in animals exposed to preconditioning followed by tMCAO. In these two experimental conditions, striatal ATP content is very low and protein synthesis is likely slowed down [13]. In support of this hypothesis, the p-AKT inhibitor, LY294002, was unable to induce a downregulation of NCKX2 expression in this brain region. Accordingly, immunofluorescence experiments did not detect an upregulated colocalization between NCKX2 and pAKT in striatal cells following preconditioning, and pAKT immunosignal emerged in intensely stained fiber bundles not expressing NCKX2. These findings suggested that a post-translational event may regulate NCKX2 expression in the striatum instead of a transcriptional pathway. With regard to potential mechanisms responsible for this phenomenon during preconditioning, it should be underlined that we have recently demonstrated that NCKX2 downregulation occurring after brain ischemia is ascribed to its cleavage by the proteolytic enzyme calpain, which is activated during anoxia. In fact, stroke-induced NCKX2 downregulation was prevented by treatment with the calpain inhibitor calpeptin [12]. In the present paper, we evaluated calpain expression in the striatum area of rats subjected to tMCAO and preconditioning and our results showed that, while calpain was dramatically activated after tMCAO, its activation was prevented by ischemic preconditioning. To verify whether the NCKX2 downregulation occurring during brain ischemia was due to its increased degradation by calpain, whose activation was prevented by preconditioning, we analyzed the effects of the calpain inhibitor, calpeptin, on NCKX2 expression in the striatum of rats exposed to transient focal cerebral ischemia. In line with our previous results [12], calpeptin, icv administered before tMCAO induction, completely prevented NCKX2 downregulation from occurring in the striatum after tMCAO. On the other hand, during preconditioning, striatal neurons can not react to hypoxia by increasing the synthesis of neuroprotective proteins, but they can decrease protein degradation since this mechanism requires less energy consumption.

The preserved expression of NCKX2 induced by preconditioning in an area such as the striatum, directly affected by ischemic damage, further underlines the key role exerted by NCKX2 in regulating sodium and calcium homeostasis in this area [9]. Indeed, thanks to its electrophysiological properties, NCKX2 can still be operative in the ischemic striatum area after hypoxic stimulus, where it may play a relevant role. In fact, this K^+^-dependent exchanger isoform may be only activated when extracellular K^+^ concentrations overcome the physiological levels being provided with a Kd of 40mM [14,15]. Interestingly, in the ischemic core, extracellular K^+^ concentrations reach such levels; therefore, these conditions are in favor of an NCKX2 activation in this brain region [15,16].

On the other hand, the neuroprotective role of NCKX2 in brain ischemia [12] is further supported by the results of the present study in which the neuroprotective effect exerted by preconditioning on tMCAO was prevented by the genetic ablation of nckx2, thus suggesting that the presence of NCXK2 renders the brain more resistant to brain ischemic damage.

Collectively, our results suggest that NCKX2 represents a new potential target to be investigated in the neuroprotective mechanisms mediated by ischemic preconditioning and support the idea that the development of new compounds able to activate NCKX2 might be a reasonable strategy for therapeutic intervention during cerebral ischemia.

## 4. Materials and Methods

### 4.1. Experimental Groups

One hundred and eight male adult Sprague–Dawley rats (Charles River) weighing 250 to 300 g and 36 nckx2+/+ and nckx2−/− C57 male 8-week-old mice weighing 22–24 g were housed under diurnal lighting conditions (12h darkness/light). It has been calculated that about 20% of the animals used had been excluded from the experimental groups due to the absence of ischemic lesions or t mortality related to the experimental procedure. Experiments were performed according to the international guidelines for animal research and approved by the Animal Care Committee of “Federico II”, University of Naples, Italy.

### 4.2. Focal Ischemia

Transient focal ischemia was induced, as previously described [17], by occlusion of the middle cerebral artery (MCA) in male rats and male mice anesthetized using 1.5% sevoflurane, 70% N_2_O and 28.5% O_2_. Briefly, a 2-O surgical monofilament nylon suture (Doccol, CA, USA) for rats or a 5-O surgical monofilament nylon suture (Doccol, CA, USA) for mice, was inserted through the external carotid artery stump and advanced into the left internal carotid artery until it blocked the origin of the middle cerebral artery. Control sham-operated animals were subjected to the same surgical procedure without insertion of the filament. Regional cerebral blood flow in the area of the right MCA was monitored in order to confirm the achievement of ischemia. Cerebral blood flow was monitored through a disposable microtip fiber optic probe (diameter 0.5mm) connected through a Master Probe to a laser Doppler computerized main unit (PF5001; Perimed, Sweden) and analyzed using PSW Perisoft 2.5 [5]. Animals that did not show a cerebral blood flow reduction of at least 70% were excluded from the experimental group, as well as animals that died after ischemia induction. In some animals, a catheter was inserted into the femoral artery to measure arterial blood gases with a blood gas analyzer before and after the surgical procedure (Rapid lab 860; Chiron Diagnostic). Rectal temperature was maintained at 37 ± 0.5 °C with a thermostatically controlled heating pad and lamp. All surgical procedures were performed under an operating stereomicroscope. After 100 min MCA occlusion in rats or 60 min in mice, the animals were re-anesthetized and the filament was withdrawn in order to restore blood flow [17,18]

### 4.3. Preconditioning Experimental Protocol

Ischemic preconditioning was induced as previously described [5]. Briefly, in rats, preconditioning was induced by a subliminal 30 min MCAO, followed by 72 h of reperfusion and 100 min of harmful MCAo. In mice, preconditioning was induced through a subliminal 15 min MCAO, followed by 72 h of reperfusion and 60 min of harmful MCAo. All animals were then recovered for 24 h [19]. The success of the experimental procedures was confirmed by measuring CBF in all the experimental steps.

### 4.4. Evaluation of the Infarct Volume

Rats were decapitated at different time intervals from tMCAO or preconditioning + tMCAO, whereas mice were decapitated 24 h after ischemia induction. The ischemic volume was evaluated by 2,3,5-triphenyl tetrazolium chloride (TTC) staining. The brains were cut into 1 mm coronal slices with a vibratome (Campden Instrument, Loughborough, UK, 752M). Sections were incubated in 2% TTC for 20 min and in 10% formalin overnight. The infarction area was calculated with image analysis software (Image-Pro Plus) [20]. To avoid a possible overestimation of the infarct volume due the presence of edema formation, the total infarct volume was expressed as a percentage of the volume of the hemisphere ipsilateral to the lesion [21].

### 4.5. Western Blotting Analysis

Cortical temporoparietal and striatum samples were harvested from rats subjected to 100 min of ischemia or to 30 min of preconditioning followed by 100 min of ischemia. These brain samples were obtained at different reperfusion times after the last occlusion: (a) 5 h; (b) 24 h; and (c) 72 h. The same group of brain samples were obtained from brains of sham-operated animals.

Rat brain samples were homogenized using an 18-gauge needle in a lysis buffer (50 mmol/L Tris–HCl, pH 7.5, 100 mmol/L NaCl, 1% Triton X-100, 0.2 mmol/L Sodium orthovanadate containing a protease inhibitor cocktail consisting of aprotinin, leupeptin and pepstatin), (Roche Diagnostic, Monza, Italy). After centrifugation at 13,400 rpm at 4 °C for 20 min, the supernatants were collected. Protein concentration was estimated using the Bradford reagent (Bio-Rad Laboratories, Segrate, Milan, Italy) Then, 100 μg of protein was mixed with a Laemmli sample buffer. The samples were separated on 8% sodium dodecyl sulfate polyacrylamide gel electrophoresis and transferred onto Amersham™ Hybond™-ECL nitrocellulose membranes (GE Healthcare, Milan, Italy). The non-specific binding sites were blocked with an incubation of 5% non-fat dry milk in Tris-buffered saline (TBS) 0.1% Tween 20 (Sigma-Aldrich, Milan, Italy) for 2 h at room temperature. Blots were then probed with antibodies to NCKX2 (1:250; Abcam, #ab192419, Cambridge, MA, USA) and β-actin (1:1000; Sigma-Aldrich, Milan, Italy), or to Calpain (1:250, Santa Cruz) diluted in Tris-buffered saline (TBS) 0.1% Tween 20 overnight (4 °C). Blots were then incubated with a horseradish peroxidase-conjugated secondary antibody (1:2000; GE Healthcare, Milan, Italy) for 60 min at room temperature in 5% non-fat dry milk and detected using an enhanced luminescence kit (GE Healthcare, Milan, Italy) [12,22].

### 4.6. Immunocytochemistry

Immunostaining and confocal immunofluorescence procedures were performed as previously described [23]. Rats were anesthetized with chloral hydrate (300 mg/kg, intraperitoneally) and perfused transcardially with 4% paraformaldehyde and 15% picric acid in phosphate buffer. The brains were sectioned coronally at 60 mm on a vibratome. After blocking, sections were incubated with the following primary antisera: mouse monoclonal anti-NCKX2 (1:500), mouse monoclonal antiphospho-AKT Ser473 (1:500, USBiological, MA, USA) and rabbit polyclonal antiphospho-AKT Ser473 (1:200, Cell Signalling Technology, MA, USA). Sections processed for immunofluorescence analysis were incubated with the corresponding fluorescent-labeled secondary antibodies (Alexa 488/Alexa 594-conjugated antimouse/antirabbit IgGs). Images were observed using a Zeiss LSM510 META/laser scanning confocal microscope. Single images were taken with an optical thickness of 0.7 m and a resolution of 1024 × 1024. In double-labeled sections, the pattern of immunoreactivity for both antigens was identical to that seen in single-stained material. Controls of the methods in the double immunofluorescence experiments included replacement of the primary antisera with normal serum (1:200). In double immunolabeling experiments, to control for a possible cross-reactivity between IgGs, some sections were processed through the same immunocytochemical sequence, except that primary antisera were replaced with normal serum, or only one primary antibody was applied, but the full complement of secondary antibodies was maintained. In addition, the secondary antibodies used were highly pre-adsorbed to the IgGs of numerous species. Tissue labeling without primary antibodies was also tested to exclude autofluorescence. No specific staining was observed under these control conditions, thus confirming the specificity of the immunosignals.

### 4.7. p-AKT and Calpain Inhibition

To assess if the changes in NCKX2 expression were mediated by a translational or by a post-translational mechanism, the effect of the p-AKT inhibitor, LY294002, and of the calpain inhibitor, calpeptin, on NCKX2 expression was evaluated in rats exposed to ischemia or preconditioning stimulus. LY294002 (5 μL, 10 μmol/L in 3% dimethyl sulfoxide) was intracerebroventricularly injected 15 min before preconditioning induction. Calpeptin (150 μg/Kg) was intracerebroventricularly injected 1 h before the beginning of ischemia. Both compounds were injected in the right lateral ventricle using the following coordinates from the bregma: anteroposterior, −0.4; laterolateral, −2.0; depth, −2.5 [24]. NCKX2 expression was evaluated in the cortex and striatum by Western Blot 72 h after treatments.

### 4.8. Statistic Analysis

Values are expressed as means ± S.E.M. Statistical analysis was performed with 2-Way ANOVA, followed by Newman–Keuls test. Neurologic deficit data were analyzed by the nonparametric Kruskal–Wallis test, followed by the Nemenyi test for the nonparametric multiple comparison. Statistical significance was accepted at the 95% confidence level (*p* < 0.05).

## Data Availability

Not applicable.
